# Supplementary material for: Multi-omics analysis identifies RFX7 targets involved in tumor suppression and neuronal processes
Source: Cell Death Discov. 2023 Mar 3;9:80. doi: 10.1038/s41420-023-01378-1 (PMC9981735; doi:10.1038/s41420-023-01378-1)

Schwab *et al.*, Supplementary Figure S1

a

uncropped immunoblots corresponding to Figure 1a

RFX7

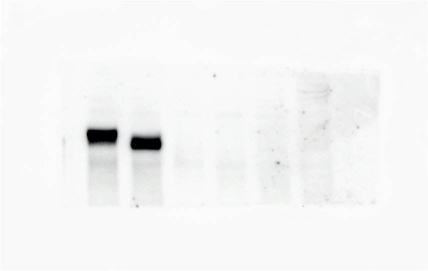

p53 and actin

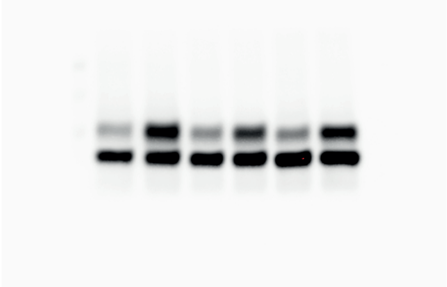

PIK3IP1

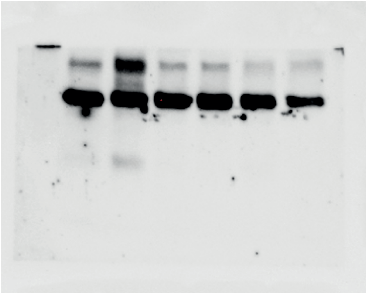

PDCD4

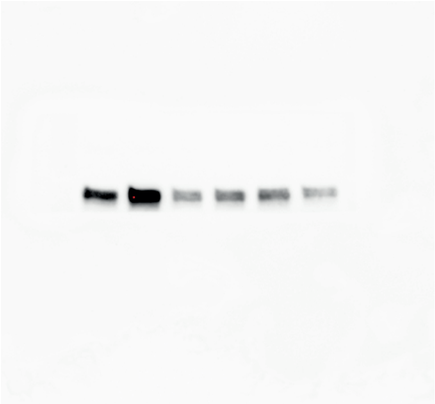

b

uncropped immunoblots corresponding to Figure 5a

RFX7

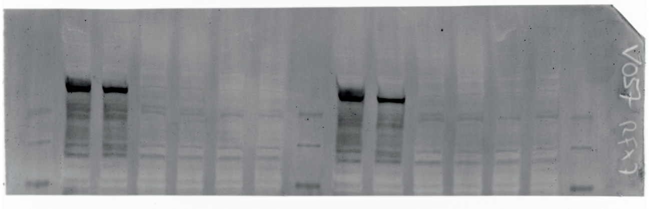

p53 and actin

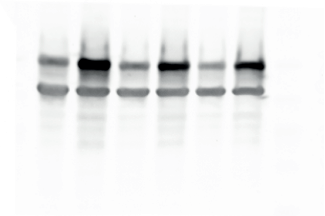

PIK3IP1

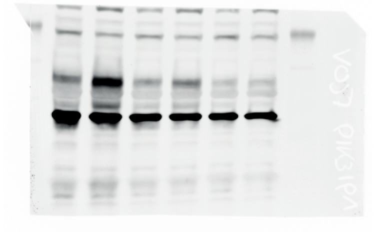

PDCD4

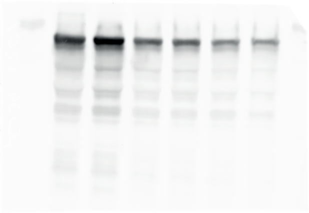

Supplement: Supplementary file 3 — Figure S1 - uncropped western blot images [file 41420_2023_1378_MOESM3_ESM.pdf]
